# Associations between Adverse Childhood Experiences and Obesity in a Developing Country: A Cross-Sectional Study among Middle-Aged and Older Chinese Adults

**DOI:** 10.3390/ijerph19116796

**Published:** 2022-06-02

**Authors:** Li Lin, Weiqing Chen, Weidi Sun, Minyan Chen, Jinghua Li, Jichuan Shen, Vivian Yawei Guo

**Affiliations:** 1 Department of Epidemiology, School of Public Health, Sun Yat-sen University, Guangzhou 510080, China; linli58@mail2.sysu.edu.cn (L.L.); chenwq@mail.sysu.edu.cn (W.C.); sunwd5@mail2.sysu.edu.cn (W.S.); chenminyan1988@163.com (M.C.); 2 Department of Health Education, Guangzhou Center for Disease Control and Prevention, Guangzhou 510440, China; shenjic@163.com; 3 Department of Biostatistics, School of Public Health, Sun Yat-sen University, Guangzhou 510080, China; lijinghua3@mail.sysu.edu.cn

**Keywords:** adverse childhood experiences, general obesity, central obesity, developing country, Chinese adults

## Abstract

Background: The association between adverse childhood experiences (ACEs) and obesity in developing countries has been underexplored and inconsistent. Methods: This cross-sectional study used data of 10,054 adults aged ≥ 45 years from the China Health and Retirement Longitudinal Study. Information on 12 ACE indicators was collected via questionnaires. General obesity was defined as a body mass index (BMI) of ≥28 kg/m². Central obesity was defined as a waist circumference of ≥90 cm for males and ≥85 cm for females. Logistic and linear regression analyses were conducted to evaluate the association of ACEs with general obesity, central obesity, BMI, and waist circumference where appropriate. Results: Compared to the non-exposed group, the experience of ≥3 ACEs was significantly associated with decreased risks of general obesity (OR = 0.83, 95% CI: 0.69, 0.999), central obesity (OR = 0.88, 95% 0.77, 0.997), and smaller BMI (β = −0.27, 95% CI: −0.50, −0.04) and waist circumference (β = −0.89, 95% CI: −1.52, −0.26). Compared to the high socioeconomic status (SES) group, such associations were more evident in those with a low SES, except for central obesity. Conclusion: ACEs were shown to be inversely associated with later-life obesity in China, especially in socioeconomically disadvantaged populations. The context-specific impacts reflect divergent roles of socioeconomic position in the obesity epidemic between developed and developing countries. Further investigations are needed to confirm whether physical activity could shift the direction of this association.

## 1. Introduction

With the unprecedented development of urbanization and the expanding obesogenic environment over the past few decades in China, increasing sedentary behaviors and over-nutrition have contributed to the nationwide obesity epidemic in both urban and rural areas [1,2]. During the 2004–2018 period in China, the prevalence of general obesity, defined with the body mass index (BMI), rose from 3.5% to 8.8% among urban residents and from 2.9% to 7.6% in rural settings [2]. Although BMI is an acceptable measure of overall obesity and has been well-established to be associated with increased risks of both morbidity and mortality [3], it is generally accepted that this indicator alone cannot reflect the distribution of body fat [4]. In contrast, central obesity characterized by an enlarged waist circumference has been considered as a surrogate of visceral adiposity [4], which is linked to greater cardiometabolic risks independent of BMI [5]. 

Typical risk factors for both general and central obesity include the excessive intake of calories, physical inactivity, sedentary behavior, and stress [6,7,8]. Several studies have also demonstrated a significant link between socioeconomic status (SES) and the risk of obesity [9,10]. However, a large body of evidence has confirmed substantial variations in this association between developed and developing countries. In developed countries, people in lower SES groups have been found to have a higher risk of obesity, while in developing countries, those in higher SES groups were more likely to be obese [11,12,13]. As the largest developing country in the world, China has shown a significant SES gap in the prevalence of obesity, with people of socially advantaged classes at a higher risk of obesity [14]. Furthermore, an urban–rural disparity in the obesity prevalence has also been observed in China [2], possibly due to the different levels of socioeconomic development between urban and rural areas. 

In addition to the above-mentioned risk factors of obesity, emerging evidence from developed countries has consistently demonstrated a positive association between exposure to adverse childhood experiences (ACEs) and adulthood obesity [15,16,17]. Nevertheless, such association in developing countries has been underexplored, with mixed findings [18,19,20]. For example, data from the Ribeirao Preto Cohort Study (RPCS) showed that a low childhood SES was associated with a lower BMI among males aged between 23 and 25 years but not in females of similar age [18]. Another cross-cohort study suggested a dose–response relationship between increasing numbers of ACEs and larger BMI and waist circumference values among British adolescents, whilst inverse but insignificant associations were found in Brazilian adolescents [19]. In contrast, a previous study among Mexican women indicated that the experience of four or more ACEs was associated with higher odds of obesity [21], similar to conclusions from studies conducted in developed countries [15,16,17]. These inconsistent findings suggested the need for further research on this topic among developing countries, especially in China, where ACEs are more prevalent [22,23]. 

Using data from the China Health and Retirement Longitudinal Study (CHARLS), we aimed to evaluate associations between cumulative ACE exposures with risks of general obesity and central obesity, as well as continuous BMI and waist circumference among middle-aged and older Chinese adults. Sensitivity analyses were further conducted for different SES groups based on current economic status and area of residence.

## 2. Materials and Methods

### 2.1. Study Design and Population

The CHARLS was a nationally representative survey with participants aged 45 years or older intended to serve the needs of scientific research on healthy ageing. Detailed information of the study design was previously published [24]. In general, participants of CHARLS were recruited from 450 villages/urban communities of 28 provinces across China using a stratified multistage probability sampling strategy. The baseline survey was conducted from June 2011 to March 2012, with subsequent follow-up surveys every two years. The 2014 life history survey additionally collected information on early life experiences. Data of this cross-sectional study were extracted from the 2014 and 2015 CHARLS surveys, as the most updated physical assessment was conducted in 2015. 

**Figure 1:**
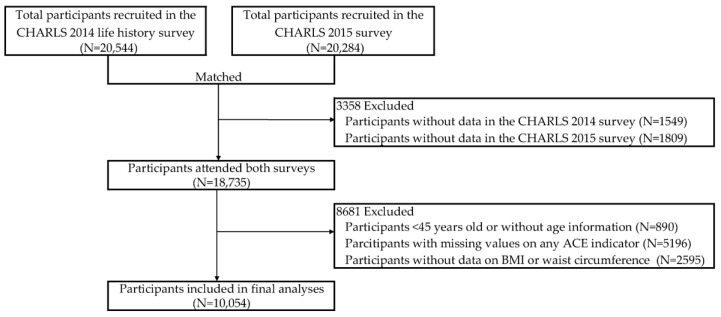
Study flowchart of participant selection.

Of the 20,544 participants interviewed during the CHARLS 2014 life history survey and the 20,284 participants recruited in the 2015 survey, 18,735 had participated in both surveys (Figure 1). After the further exclusion of participants aged below 45 years or those without age information (N = 890), individuals without data on any ACE indicators (N = 5196), and those without information on BMI or waist circumference (N = 2595), a total of 10,054 eligible participants were included in final analyses.

Ethical approval for this study was obtained from the Institutional Review Board (IRB) at Peking University (IRB approval numbers: IRB00001052-11015 and IRB00001052-11014.14). Written informed consent was signed by each respondent involved in the survey.

### 2.2. Definition of Adverse Childhood Experiences

Based on previous literature [25,26,27], a total of 12 stressful life events that occurred before the age of 17 years old were measured as ACE indicators: physical abuse, emotional neglect, household substance abuse, household mental illness, domestic violence, incarcerated household member, parental separation or divorce, unsafe neighborhood, bullying, parental death, sibling death, and parental disability. Detailed questionnaire items and definitions of individual ACE exposure are listed in Supplementary Table S1. Responses to each ACE indicator were dichotomized as yes (coded as 1) or no (coded as 0). A cumulative ACE score ranging from 0 to 12 was generated by summing the 12 ACE indicators for each participant. We further grouped participants into four categories based on the cumulative ACE score, i.e., 0, 1, 2, and ≥3 ACEs.

### 2.3. Measurements of Obesity

Standing height and weight were measured with a stadiometer (SecaTM 213, Hangzhou, China) and a scale (OmronTM HN-286, Yangzhou, China), respectively. BMI was calculated as weight (kg) divided by the square of height (m^2^). Based on the recommended standard for Chinese adults, participants with a BMI of ≥ 28 kg/m^2^ were defined as general obesity [28,29].

Waist circumference was assessed with a soft measuring tape around the abdomen at the level of the navel while participants held their breath at the end of an exhalation. Central obesity was defined as a waist circumference of ≥90 cm in males and ≥85 cm in females [30]. 

### 2.4. Covariates

Data on demographic characteristics and lifestyle factors, including age, sex, ethnicity, educational level, current marital status, area of residence, current economic status, smoking status, drinking status, and nighttime sleep duration, were collected through face-to-face interviews. Ethnicity was classified into Han ethnicity and the ethnic minority population, as Han is the largest ethnic group in China. Educational level was categorized into illiterate or no formal education, primary school, and middle school or above. Current marital status was grouped into married/cohabitated and unmarried. The latter included separated, divorced, widowed, and never married. Area of residence was divided into urban and rural areas according to the criteria of National Bureau of Statistics of China [31]. Current economic status was measured by annual per-capita household consumption expenditure, which has been demonstrated to be a better indicator to capture standards of living than income [32]. Household consumption expenditures (excluding medical expenditures) consisted of household spending on food, communication, transportation, clothing, durable goods, etc., over the past year, which was added up and divided by household size to calculate per-capita expenditure [33]. Low economic status was defined as the lowest tertile of the annual per-capita household consumption expenditure; otherwise, participants were categorized into the group with relatively high economic status. An SES indicator was further generated based on area of residence and current economic status. Participants were grouped into the low SES group if they lived in rural area or had a low economic status, while urban residents of high economic status were classified into the high SES group. Smoking and drinking status were grouped into never users versus ever users. Nighttime sleep duration in hours was measured with self-reports, based on which participants were grouped into three categories, i.e., ≤6 h, 6–8 h, and >8 h per night [34].

### 2.5. Statistical Analysis 

Descriptive characteristics are presented as mean ± standard deviation (SD) for continuous data and frequency (percentage) for categorical data. Comparisons of variables between general obesity groups or central obesity groups were performed using independent student t-tests for continuous variables and Chi-square tests for categorical characteristics. 

Logistic regression models were established to assess the impact of ACEs on categorical outcomes (i.e., general obesity and central obesity), while linear regression analyses were conducted when the outcomes were continuous BMI and waist circumference. Model 1 was a crude model. Model 2 was adjusted for age, sex, ethnicity, educational level, current marital status, area of residence, current economic status, smoking status, drinking status, and nighttime sleep duration. Linearity, normality, homoscedasticity and absence of multicollinearity were examined for all linear regression models. Sensitivity analyses were further conducted for different SES groups, with adjustment for the same confounders included in model 2 except for area of residence and current economic status. 

All analyses were performed using Stata 15.0 (StataCorp. College Station, TX, USA). All tests were two-sided, and a *p*-value of less than 0.05 was considered to be statistically significant.

## 3. Results

The study population comprised 10,054 participants, with a mean age of 59.8 (SD: 9.5) years and 52.1% being females. The prevalence of general obesity and central obesity were 13.7% and 48.6%, respectively (Table 1). Compared to participants without general obesity, those with BMI values of 28 kg/m^2^ or above were younger, more educated, and more likely to be females, ethnic minorities, urban residents, currently married/cohabitated, and never smokers or never drinkers. Regarding the comparison of central obesity status, results were similar to that of general obesity status, though participants with central obesity were more likely to have a high economic status than those without. In addition, the characteristic of ethnicity and current marital status were comparable between different central obesity groups. We also found that participants with either type of obesity tended to have relatively lower level of ACE exposure compared to their non-obese counterparts.

The associations of ACEs with general and central obesity are presented in Table 2. In the crude model, compared to participants without any ACE exposure, those with the experience of three or more ACEs had 25% (95% CI: 12%, 36%) and 19% (95% CI: 9%, 28%) lower risks of general obesity and central obesity, respectively. After adjustment for confounders in model 2, the magnitude of estimated ORs was slightly attenuated but remained significant (OR = 0.83, 95% CI: 0.69, 0.999 for general obesity; OR = 0.88, 95% CI: 0.77, 0.997 for central obesity). Regarding continuous measures, exposure to three or more ACEs was significantly associated with smaller BMI (β = −0.45, 95% CI: −0.66, −0.23) and waist circumference (β = −1.05, 95% CI: −1.63, −0.47) values than those without experience of ACEs in model 1. In the adjusted model 2, individuals with the experience of ≥3 ACEs were still shown to have smaller BMI and waist circumference values compared to the reference group (β = −0.27, 95% CI: −0.50, −0.04 for BMI; β = −0.89, 95% CI: −1.52, −0.26 for waist circumference).

Table 3 and Table 4 show the results of sensitivity analyses for different SES groups. The inverse association between three or more ACEs and the risk of general obesity remained significant in the low SES group (OR = 0.79, 95% CI: 0.63, 0.98), but such an association was not evident for participants of the high SES group (OR = 0.96, 95% CI: 0.69, 1.34). Furthermore, although the experience of three and more ACE exposures was associated with lower odds of central obesity than those without in both the low and high SES groups, the effect estimates did not reach statistical significance in either subgroup. In terms of continuous outcomes, the experience of three or more ACEs was only significantly associated with smaller BMI (β = −0.36, 95% CI: −0.63, −0.09) and waist circumference (β = −0.97, 95% CI: −1.70, −0.24) values in the low SES group, not in the high SES group (β = 0.00, 95% CI: −0.44, 0.44 for BMI; β = −0.59, 95% CI: −1.81, 0.64 for waist circumference).

## 4. Discussion

In the present study, exposure to three or more ACEs was found to be associated with decreased risks of general obesity and central obesity, as well as smaller BMI and waist circumference values, compared to a non-exposed group. In addition, compared to individuals of a high SES, the inverse association between ACEs and adiposity measures was relatively more evident in the low SES group, except for central obesity.

Although the link between exposure to ACEs and an increased risk of obesity has been consistently demonstrated for adults in developed countries [15,16,17], evidence regarding this association in developing world has been limited and inconsistent [18,19,20,21]. One study supporting our findings was conducted in Brazil and showed that a low SES during childhood was associated with a lower risk of obesity in males during their early adulthood, as opposed to results observed in females [18]. Another cross-country study with adolescents from Brazil also showed an inverse but insignificant association between ACEs and obesity [19]. However, data from Mexican women have demonstrated a positive association between ACEs and the risk of adulthood obesity [21], similar to that found in developed countries [15,16,17]. The disparities across studies could be explained by differences of population characteristics and ACE measurements. Previous studies have suggested that the association between ACEs and obesity could substantially vary across different racial or ethnic groups [35,36]. Additionally, the perception of ACEs is culture-specific, which may influence ACE reporting and the response to ACE exposure [19,37]. For example, among older generations in China, minor physical punishment against children has been considered acceptable and even beneficial. While participants in our study were relatively older, the inconsistent findings across different studies are plausible. In addition to our study, the long-term impact of ACEs on obesity risk deserves further investigation in developing regions, especially in the Eastern world where socio-cultural backgrounds and life patterns are more homogeneous.

In contrary to studies conducted in developed countries, we observed significantly lower risks of both general and central obesity in individuals with experiences of ≥3 ACEs. It is well-accepted that early life stress originated from ACEs could elevate the level of allostatic load and chronic inflammation, leading to the dysregulation of immune and metabolic systems [38,39], which is one of the underlying pathways of obesity [40]. However, exposure to ACEs is also a driver of disadvantaged SES during adulthood due to, e.g., lower educational attainment and income [41,42], which could result in discrepant patterns of obesity prevalence between developing and developed countries. Specifically, in developing regions such as China, people of a lower SES may have limited resources of energy-dense foods such as fat and animal products due to unaffordability, while individuals of a higher SES may have more access to processed and calorie-rich foods [43,44]. In addition, the widespread belief that excessive weight is a symbol of prosperity in Chinese culture makes it challenging for socioeconomically advantaged population to exercise and keep a balanced diet, especially among older adults who experienced food shortage or famine in the last century [43,44,45,46]. Thus, associations between SES and obesity in developing countries might compensate and even outweigh the adverse biological changes induced by ACEs that could have led to increased adiposity. On the contrary, the over-consumption of high-calorie and nutrient-poor foods has been found to be more likely to occur in socioeconomically disadvantaged groups in developed countries, whereas those of a higher SES can afford a healthier diet and have more opportunities to exercise [47]. Accordingly, studies conducted in developed countries have consistently found a link between experience of ACEs and increased risk of obesity [15,16,17], while in the context of China, we found that ACE exposure was inversely linked to obesity rates.

In parallel to our hypothesis, we found that ACEs were linked to a decreased risk of obesity and smaller adiposity measures only in the low SES group, not in those with a high SES. Possible explanations for this discrepancy could be the divergent dietary patterns and healthy behaviors between different SES groups. Compared to individuals of a high SES, subjects of socioeconomically disadvantaged groups are more likely to reside in rural area and work in agricultural industry or labor-intensive occupations that involve more physical activities [48,49]. Additionally, a low SES population can usually only afford low-fat and nutrient-poor diets and tends to have high energy expenditure, which have been linked to lower risks of obesity [49,50]. On the contrary, urban dwellers with relatively favorable economic condition are predisposed to have higher levels of energy intake and more sedentary behaviors, subsequently contributing to their increased bodyweight and enlarged waist circumference [48,49]. However, in this study, the decreased risk of central obesity induced by ACEs was also not found to be significant in the disadvantaged SES group, which aligned with reports of more rapid increases in the prevalence of central obesity in rural residents with normal BMI values than their urban counterparts [51,52].

Notably, the social and economic context, alongside income growth, urbanization, and improved food security, has drastically changed in China during the past few decades [53]. These unprecedented shifts have led to an alarming rise of obesity rates in China, regardless of area of residence [2]. For example, with increased household income and reduced manual occupations, diet compositions have moved towards high-fat foods from animal sources and the sedentary lifestyle has become more prevalent among rural residents and those with a relatively low economic status [49,54]. Consequently, the inverse association between ACEs and the risk of adiposity found in Chinese adults might shift into the opposite direction due to the narrowing SES differences in obesity risk profiles. Therefore, prevention initiatives should be urgently implemented in order to combat the potential transition of obesity risk induced by childhood adversities.

To the best of our knowledge, this was the first study to assess the associations of ACEs with risks of general and central obesity among middle-aged and older people in China. Participants were categorized into four groups by the cumulative score of ACEs, which avoided overestimating the impact of single ACE indicator on outcomes [55]. Furthermore, we investigated such associations in different SES groups, providing evidence to facilitate public health interventions on the great transitions of obesity risk profiles in China.

Nonetheless, several limitations of this study should be discussed. First, a large proportion of participants were excluded from analyses due to missing data, which might have reduced the generalizability of this study. Cautions should be taken when interpreting our findings. Second, although the duration, frequency, or severity of ACE exposures have been demonstrated to be associated with adulthood cardiometabolic health [56], such information was not available in the CHARLS. Third, information on experiences of ACEs was collected through retrospective interviews, which might have introduced recall bias. Nevertheless, retrospective measurements of ACEs have shown good reliability and stability over time [57]. Future studies using prospective ACEs are also encouraged to confirm our findings. Last but not least, while we controlled for several risk factors of obesity, other well-established risk factors of obesity, such as physical activity and dietary habits, were not included in the analyses due to limited data or data unavailability in the CHARLS surveys [6], which might have caused residual confounding. Nevertheless, we conducted a sensitivity analysis by additionally adjusting for physical activity in regression models for participants with data on this information (N = 4900). We found that exposure to ≥3 ACEs was still associated with lower obesity risks and smaller adiposity measures, though such associations were attenuated to statistical insignificance, except for waist circumference (data not shown). However, it should be noted that physical activity was measured with the modified short-form International Physical Activity Questionnaire in CHARLS, which might have caused inaccurate estimates of physical activity and biased results regarding these associations [58]. Further studies using standardized questionnaires or objective measurements (e.g., accelerometer) to assess levels of physical activity are warranted to confirm such findings.

## 5. Conclusions

This study showed that in developing countries such as China, ACE exposure is linked to reduced risks of general and central obesity among middle-aged and older adults, which contrasted the findings reported for developed countries. We also found that such associations were relatively more evident in participants with disadvantaged socioeconomic conditions than that in high SES groups. Our findings confirmed the well-accepted disparities between developing and developed countries regarding the roles of socioeconomic gradients in the obesity epidemic. Furthermore, interventions to stop the obesity epidemic should be more rigorously promoted in China.

## Figures and Tables

**Table 1:** Comparison of characteristics by status of general and central obesity in 2015 CHARLS survey.

	General Obesity ^a^		*p*-Value	Central Obesity ^b^		*p*-Value
	Yes	No		Yes	No	
**N (%)**	1377 (13.7%)	8677 (86.3%)		4890 (48.6%)	5163 (51.4%)	
Mean age (years)	57.4 (8.5)	60.2 (9.6)	<0.001	59.3 (9.2)	60.2 (9.7)	<0.001
Sex, n (%)			<0.001			<0.001
Male	533 (38.7%)	4281 (49.3%)		1840 (37.6%)	2974 (57.6%)	
Female	843 (61.3%)	4396 (50.7%)		3050 (62.4%)	2189 (42.4%)	
Ethnicity, n (%)			0.005			0.780
Han ethnicity	1242 (90.6%)	8022 (92.7%)		4509 (92.5%)	4754 (92.4%)	
Ethnic minority population	129 (9.4%)	629 (7.3%)		365 (7.5%)	393 (7.6%)	
Educational level, n (%)			0.009			<0.001
Illiterate/No formal education	504 (41.1%)	3393 (42.9%)		1868 (42.0%)	2029 (43.3%)	
Primary school	251 (20.5%)	1824 (23.1%)		955 (21.5%)	1120 (23.9%)	
Middle school or above	470 (38.4%)	2694 (34.1%)		1628 (36.6%)	1536 (32.8%)	
Area of residence, n (%)			<0.001			<0.001
Rural	768 (55.8%)	5569 (64.2%)		2796 (57.2%)	3540 (68.6%)	
Urban	609 (44.2%)	3108 (35.8%)		2094 (42.8%)	1623 (31.4%)	
Current economic status, n (%)			0.071			<0.001
Low economic status	453 (32.9%)	3065 (35.4%)		1605 (32.9%)	1913 (37.2%)	
High economic status	923 (67.1%)	5587 (64.6%)		3275 (67.1%)	3234 (62.8%)	
Current marital status, n (%)			<0.001			0.680
Married/cohabitated	1256 (91.4%)	7587 (87.5%)		4295 (87.9%)	4548 (88.2%)	
Unmarried	118 (8.6%)	1085 (12.5%)		592 (12.1%)	611 (11.8%)	
Smoking status, n (%)			<0.001			<0.001
Never smoker	899 (67.3%)	4715 (55.8%)		3166 (66.5%)	2448 (48.7%)	
Ever smoker	437 (32.7%)	3735 (44.2%)		1593 (33.5%)	2579 (51.3%)	
Drinking status, n (%)			<0.001			<0.001
Never drinker	808 (58.9%)	4528 (52.3%)		2811 (57.6%)	2525 (49.0%)	
Ever drinker	563 (41.1%)	4136 (47.7%)		2069 (42.4%)	2630 (51.0%)	
Nighttime sleep duration, n (%)			0.084			0.125
≤6 h	4259 (50.2%)	633 (46.9%)		2553 (50.6%)	2339 (48.8%)	
6–8 h	3416 (40.2%)	577 (42.8%)		1998 (39.6%)	1995 (41.6%)	
>8 h	812 (9.6%)	139 (10.3%)		492 (9.8%)	459 (9.6%)	
BMI (kg/m^2^)	30.4 (3.0)	23.0 (2.8)	<0.001	26.6 (3.1)	21.5 (2.5)	<0.001
Waist circumference (cm)			<0.001			<0.001
Male	102.8 (7.2)	84.7 (8.9)		97.5 (6.0)	80.0 (6.2)	
Female	100.2 (7.8)	84.4 (8.6)		93.8 (7.0)	77.5 (5.5)	
Number of ACEs, n (%)			0.005			<0.001
0	299 (21.7%)	1628 (18.8%)		979 (20.0%)	948 (18.4%)	
1	386 (28.0%)	2318 (26.7%)		1331 (27.2%)	1373 (26.6%)	
2	299 (21.7%)	1883 (21.7%)		1104 (22.6%)	1077 (20.9%)	
≥3	393 (28.5%)	2848 (32.8%)		1476 (30.2%)	1765 (34.2%)	

Abbreviation: ACE, adverse childhood experience; BMI, body mass index. Continuous data are reported as mean ± SD, and categorical data are reported as frequency (percentage). ^a^ General obesity was defined as a BMI of ≥ 28 kg/m^2^. ^b^ Central obesity was defined as a waist circumference of ≥90 cm in males and ≥85 cm in females.

**Table 2:** Associations between the number of ACEs and general obesity, central obesity, BMI, and waist circumference.

	General Obesity ^a^	Central Obesity ^b^	BMI	Waist Circumference
**Model 1 ^c^**	OR (95% CI)	OR (95% CI)	β (95% CI)	β (95% CI)
**Number of ACEs**				
0	1 [reference]	1 [reference]	1 [reference]	1 [reference]
1	0.91 (0.77, 1.07)	0.94 (0.84, 1.06)	−0.12 (−0.35, 0.10)	−0.16 (−0.76, 0.44)
2	0.86 (0.73, 1.03)	0.99 (0.88, 1.12)	−0.11 (−0.34, 0.13)	−0.14 (−0.77, 0.50)
≥3	0.75 (0.64, 0.88) *	0.81 (0.72, 0.91) *	−0.45 (−0.66, −0.23) *	−1.05 (−1.63, −0.47) *
**Model 2 ^d^**				
**Number of ACEs**				
0	1 [reference]	1 [reference]	1 [reference]	1 [reference]
1	0.93 (0.78, 1.11)	0.93 (0.81, 1.06)	−0.12 (−0.35, 0.11)	−0.34 (−0.98, 0.31)
2	0.99 (0.82, 1.20)	1.05 (0.91, 1.20)	0.04 (−0.21, 0.28)	−0.05 (−0.73, 0.62)
≥3	0.83 (0.69, 0.999) *	0.88 (0.77, 0.997) *	−0.27 (−0.50, −0.04) *	−0.89 (−1.52, −0.26) *

Abbreviation: ACE, adverse childhood experience; BMI, body mass index; OR, odds ratio; CI, confidence interval. ^a^ General obesity was defined as a BMI of ≥28 kg/m^2^. ^b^ Central obesity was defined as a waist circumference of ≥90 cm in males and ≥85 cm in females. ^c^ Model 1 was a crude model. ^d^ Model 2 was adjusted for age, sex, ethnicity, educational level, current marital status, area of residence, current economic status, smoking status, drinking status, and nighttime sleep duration. * *p* < 0.05.

**Table 3:** Associations of the number of ACEs with general and central obesity in different SES groups.

	General Obesity ^a^		Central Obesity ^b^	
	Low SES ^c^	High SES	Low SES	High SES
**Number of ACEs**	OR (95% CI)	OR (95% CI)	OR (95% CI)	OR (95% CI)
0	1 [reference]	1 [reference]	1 [reference]	1 [reference]
1	0.87 (0.70, 1.08)	1.13 (0.81, 1.56)	0.93 (0.79, 1.08)	0.97 (0.76, 1.25)
2	0.89 (0.71, 1.12)	1.24 (0.88, 1.75)	1.03 (0.88, 1.21)	1.10 (0.85, 1.44)
≥3	0.79 (0.63, 0.98) *	0.96 (0.69, 1.34)	0.88 (0.76, 1.03)	0.88 (0.69, 1.13)

Abbreviation: ACE, adverse childhood experience; SES: socioeconomic status; OR, odds ratio; CI, confidence interval. ^a^ General obesity was defined as a BMI of ≥28 kg/m^2^. ^b^ Central obesity was defined as a waist circumference of ≥90 cm in males and ≥85 cm in females. ^c^ SES was measured by area of residence and current economic status. A low SES was defined if participants lived in a rural area or had a low economic status, while the high SES group comprised urban residents of high economic status. The models were adjusted for age, sex, ethnicity, educational level, current marital status, smoking status, drinking status, and nighttime sleep duration. * *p* < 0.05.

**Table 4:** Associations of the number of ACEs with BMI and waist circumference in different SES groups.

	BMI		Waist Circumference	
	Low SES ^a^	High SES	Low SES	High SES
**Number of ACEs**	β (95% CI)	β (95% CI)	β (95% CI)	β (95% CI)
0	1 [reference]	1 [reference]	1 [reference]	1 [reference]
1	−0.22 (−0.50, 0.05)	0.25 (−0.19, 0.69)	−0.45 (−1.20, 0.30)	0.29 (−0.94, 1.51)
2	−0.11 (−0.39, 0.18)	0.43 (−0.03, 0.90)	−0.30 (−1.09, 0.49)	0.61 (−0.68, 1.91)
≥3	−0.36 (−0.63, −0.09) *	0.00 (−0.44, 0.44)	−0.97 (−1.70, −0.24) *	−0.59 (−1.81, 0.64)

Abbreviation: ACE: adverse childhood experience; SES: socioeconomic status; BMI: body mass index; CI, confidence interval. ^a^ SES was measured by area of residence and current economic status. A low SES was defined if participants lived in a rural area or had a low economic status, while the high SES group comprised urban residents of high economic status. The models were adjusted for age, sex, ethnicity, educational level, current marital status, smoking status, drinking status, and nighttime sleep duration. * *p* < 0.05.

## Data Availability

The data underlying this article are available in a public, open access repository, and can be accessed at China Health and Retirement Longitudinal Study (CHARLS) http://charls.pku.edu.cn/en/ (accessed on 15 September 2020).

## Supplementary Materials

The following supporting information can be downloaded at: https://www.mdpi.com/article/10.3390/ijerph19116796/s1, Table S1: Questionnaire items and definitions of each ACE indicator.
