# Supplementary material for: Associations between Adverse Childhood Experiences and Obesity in a Developing Country: A Cross-Sectional Study among Middle-Aged and Older Chinese Adults
Source: Int J Environ Res Public Health. 2022 Jun 2;19(11):6796. doi: 10.3390/ijerph19116796 (PMC9180433; doi:10.3390/ijerph19116796)
Supplement: Supplementary file 1 [file ijerph-19-06796-s001.zip › ijerph-1726684-supplementary.pdf]

Supplementary Table S1. Questionnaire items and definitions of each ACE indicator.

ACEs Indicators	Questionnaire Items
**Physical abuse**	When you were growing up, did your female/male guardian ever hit you? (often[a], sometimes[a], rarely, or never)
**Emotional neglect**	How much love and affection did your female guardian give you while you were growing up? (often, sometimes, rarely[a], or never[a])
	How much effort did your female guardian put into watching over you? (a lot, some, a little[a], or not at all[a])
**Household substance abuse**	During the years you were growing up, did your female/male guardian ever have alcoholism or drug? (yes[a] or no)
**Household mental illness**	Did your female/male guardian have abnormality of mind when you were young? (yes[a] or no)
	During the years you were growing up, had your female/male guardian often showed continued signs of sadness or depression? (during all[a], most[a], some, or only a little of the childhood)
**Domestic violence**	Have your father/mother ever beat up your mother/father? (often[a], sometimes[a], not very often, or never)
**Incarcerated household member**	During the years you were growing up, have your female/male guardian ever been arrested or sent to prison? (yes[a] or no)
**Parental separation or divorce**	Were your biological parents divorced (including long separation due to emotional problems) before you were 17 years? (yes[a] or no)
**Unsafe neighborhood**	Was it safe being out alone at night in the neighborhood where you lived as a child? (very safe, somewhat safe, not very safe[a], or not safe at all[a])
**Bullying**	When you were a child, how often were you picked on or bullied by kids in your neighborhood? (often[a], sometimes[a], not very often, or never)
	When you were a child, how often were you picked on or bullied by kids in your school? (often[a], sometimes[a], not very often, or never)
**Parental death[b]**	Either of the parents was dead before participant was 17 years. (yes[a] or no)
**Sibling death[c]**	Any of the siblings was dead before participant was 17 years. (yes[a] or no)
**Parental disability**	Did your female/male guardian have a long time being sick on bed when you were young? (yes[a] or no)
	Did your female/male guardian have a serious deformity when you were young? (yes[a] or no)

Abbreviation: ACEs: Adverse Childhood Experiences.

[a] Answers indicate thresholds for ACEs.

[b] Calculated based on dates of birth and their parental death

[c] Calculated based on dates of birth and their sibling death
